# Integrating remotely sensed surface water extent into continental scale hydrology

**DOI:** 10.1016/j.jhydrol.2016.10.041

**Published:** 2016-12

**Authors:** Beatriz Revilla-Romero, Niko Wanders, Peter Burek, Peter Salamon, Ad de Roo

**Affiliations:** a European Commission, Joint Research Centre, Ispra, Italy; b Utrecht University, Faculty of Geosciences, Utrecht, The Netherlands; c Department of Civil and Environmental Engineering, Princeton University, United States; d International Institute of Applied Systems Analysis, Laxenburg, Austria

**Keywords:** Data assimilation, Ensemble Kalman filter (EnKF), Global Flood Detection System (GFDS), LISFLOOD model, Continental hydrology, Surface water

## Abstract

In hydrological forecasting, data assimilation techniques are employed to improve estimates of initial conditions to update incorrect model states with observational data. However, the limited availability of continuous and up-to-date ground streamflow data is one of the main constraints for large-scale flood forecasting models. This is the first study that assess the impact of assimilating daily remotely sensed surface water extent at a 0.1° × 0.1° spatial resolution derived from the Global Flood Detection System (GFDS) into a global rainfall-runoff including large ungauged areas at the continental spatial scale in Africa and South America. Surface water extent is observed using a range of passive microwave remote sensors. The methodology uses the brightness temperature as water bodies have a lower emissivity. In a time series, the satellite signal is expected to vary with changes in water surface, and anomalies can be correlated with flood events. The Ensemble Kalman Filter (EnKF) is a Monte-Carlo implementation of data assimilation and used here by applying random sampling perturbations to the precipitation inputs to account for uncertainty obtaining ensemble streamflow simulations from the LISFLOOD model. Results of the updated streamflow simulation are compared to baseline simulations, without assimilation of the satellite-derived surface water extent. Validation is done in over 100 *in situ* river gauges using daily streamflow observations in the African and South American continent over a one year period. Some of the more commonly used metrics in hydrology were calculated: KGE’, NSE, PBIAS%, R^2^, RMSE, and VE. Results show that, for example, NSE score improved on 61 out of 101 stations obtaining significant improvements in both the timing and volume of the flow peaks. Whereas the validation at gauges located in lowland jungle obtained poorest performance mainly due to the closed forest influence on the satellite signal retrieval. The conclusion is that remotely sensed surface water extent holds potential for improving rainfall-runoff streamflow simulations, potentially leading to a better forecast of the peak flow.

## Highlights

• First continent-scale assimilation of surface water extent into hydrological model.
• Improvements in flood peaks timing and volume for 60% of validated gauges.
• Daily surface water extent provide promising opportunities for ungauged regions.

## 1 Introduction

Flood forecasting systems are based on rainfall-runoff, channel flow routing, or snow-melt models, at times coupled with land surface models. These models or systems aim at simulating streamflow as close as possible to reality, and *in situ* streamflow time series typically used as a reference ground “truth”. However, the use of *in situ* observational data in near real-time flood forecasting systems is constrained due to its public unavailability at near real-time in many regions of the globe. In addition, for many large rivers, even if gauge data are available, the network might be very sparse (e.g., at Niger River). As complementary data, remotely-sensed products have been recognised as very valuable (van Dijk and Renzullo, 2011), having particular potential for use within sparsely equipped and ungauged regions where these remotely-sensed products are the only observations available. Further, remote sensing data are very useful as they provide routinely collected data with a wide spatial extent and available for scientific research and applications, and their use in hydrologic forecasting needs to be fully explored. Both *in situ* and satellite-derived data are used for calibration and validation of hydrological models (e.g., Di Baldassarre et al., 2009, Gupta et al., 1998, Madsen, 2000, Wanders et al., 2014a). Other methodologies used to enhance the skill of the simulated streamflow are (a) data assimilation of observations into a model (e.g., Clark et al., 2008, Seo et al., 2009), and (b) post-processing of the hydrological ensemble predictions (e.g., Bogner and Kalas, 2008, van Andel et al., 2013). Both methodologies have diverse strengths and, up to a certain point, are complementary. Therefore, the use of both is highly recommended (Bourgin et al., 2014), if we aim to improve forecast reliability and accuracy.

Data assimilation schemes are expected to reduce hydrological uncertainty of hydrological models (Bates, 2012, Bourgin et al., 2014), especially at shorter lead times. Despite constant development in the use of data assimilation technics in operational hydrological forecasting and Earth science in general (Reichle, 2008, Seo et al., 2014), the theoretical frameworks and the adequate characterisation of uncertainties still provides important options for both challenges and opportunities alike (see Liu et al., 2012 for a review). Another research field which requires exploration is the assimilation of observations, with emphasis on those not use in developing the model. Assimilation of state variables such as streamflow (Rafieeinasab et al., 2014, Randrianasolo et al., 2014, Sun et al., 2015) and remotely-sensed soil moisture (see Kornelsen and Coulibaly, 2013, Ni-Meister, 2008 for a review) and snow products (Franz et al., 2014, Slater and Clark, 2006, Thirel et al., 2013), has progressively been tested in recent years. Whereas not so many studies have evaluated the impact of assimilating hydraulic information such as remotely-sensed surface water extent data. Some studies (see Table 1) water extents [1–6]. Other studies have also explored the possibilities of using the surface water height and inundation extent data from the future Surface Water and Ocean Topography (SWOT, https://swot.jpl.nasa.gov/) [7–8] satellite mission, showing promising applications. One study has previously also tested to directly assimilate low resolution remote sensed flood extents-as intendent in this study- into a 2-D flood model, by using MODIS derived data [9]. In addition, to the authors’ knowledge only one study has attempted to assimilate passive-microwave surface water extent changes derived from the Global Flood Detection System (GFDS) within a rainfall-runoff model derived water levels were used as a proxy for *in situ* streamflow at a specific point location, instead of using the satellite-retrieved raw spatial signal as is the objective in this study. However, most of these studies focus solely on a single river reach or catchments, and often on specific flood events due to limited temporal availability and cost of high resolution satellite imagery.flood events due to limited temporal availability and cost of high resolution satellite imagery.

Surface water extent from the Global Flood Detection System (GFDS) is observed using a range of passive microwave remote sensors. The methodology uses the brightness temperature, as water bodies have a lower emissivity. In a time series, the satellite signal is expected to vary with changes in water surface, and anomalies can be correlated with flood events. The GFDS data have been previously used for a range of applications such as estimating streamflow (Brakenridge et al., 2007, Revilla-Romero et al., 2014), river discharge nowcasting and forecasting (Hirpa et al., 2013), model calibration, validation of floods events.

We implemented a data assimilation scheme with the aim to improve the prediction of the flood peak. However, we test it using a climatology forcing as a first step, although the effects using a probabilistic meteorological forecast should be further investigated. Therefore, we examined the feasibility of using surface water changes from the Global Flood Detection System (GFDS) for data assimilation using the ensemble Kalman filter (EnKF) within a rainfall-runoff model for the African and South American basins. The aim of this proof-of-concept study is to test whether assimilation of exclusively satellite-derived surface water changes will positively impact the skill of the simulated streamflow to reproduce the hydrograph, especially during flood peaks on large (>10,000 km^2^) and slow-motion catchments. The reason that drove this decision is that there are large regions of the world ungauged, and those gauged with publicly provided real-time data are also scarce, and we wanted to design a framework also valid from those areas. Therefore, assimilating satellite-derived information into the hydrological model have an important added value for those regions where *in situ* measurements are not available; and it can be implemented independently of these datasets.

The rainfall-runoff model employed in this study is the recently upgraded LISFLOOD global version. It runs at a daily time step using the Watch WFDEI dataset as the meteorological forcing (Weedon et al., 2011). LISFLOOD Global currently incorporates a module for data assimilation which has been successfully applied using soil moisture (Wanders et al., 2014b) within the European Upper Danube catchment for the European Flood Awareness System (EFAS). However, the new set up of LISFLOOD Global used here is currently not yet incorporated within a continental or global flood forecasting system such as the Global Flood Awareness System (GloFAS).

In Section 2 we present the data used and study regions. Section 3 describes the methodologies including the model, data assimilation framework, and assessment procedures. Results and discussion are presented in Section 4, and finally conclusions are summarised in Section 5.

## 2 Data and study region

### 2.1 Study region

**Fig. 1:**
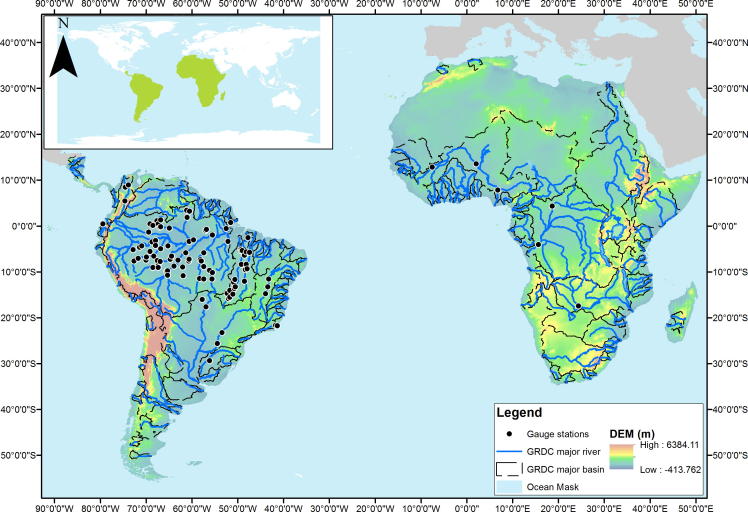
Study region. Gauging stations used for validation are represented with black dots, black dotted lines represent river basin boundaries, and blue lines represent major rivers. The background is the 10 km resolution Digital Elevation Model (DEM) used for the model. Inlet map shows the area mask of the African and South American continents used for Data Assimilation. (For interpretation of the references to colour in this figure legend, the reader is referred to the web version of this article.)

The rainfall-runoff LISFLOOD Global model was used in this study (Van Der Knijff et al., 2010). However, for testing the effects of assimilating satellite-derived surface water extent on the simulation of the streamflow, we focus on African and South American catchments due to the potential benefits that an improvement of the streamflow simulations can bring to those regions, as many of their water authorities lack of a catchment-wide hydrological model. Fig. 1 shows the studied river basins, main rivers from the Global Runoff Data Centre (GRDC), and the ground river gauges.

### 2.2 Ground streamflow data

Daily ground streamflow time series were obtained from the Global Runoff Data Centre (GRDC, 2015). For this proof-of-concept study, only one year of data was used, although the GFDS signal is available for most of the globe since 1998. We choose 2003 in order to have the largest number of *in situ* gauges data for validation, especially within the African continent. Many of these cease to either record or provide data to GRDC after 2004 due to an smaller gauging network coverage and more restricted access to national scale information (Hannah et al., 2011).

Furthermore, based on previous research of GFDS (Revilla-Romero et al., 2014) and LISFLOOD global model recommendations (Alfieri et al., 2013), our criteria was to selected only stations with a daily mean average discharge larger than 500 m^3^ s^−1^ and an upstream area larger than 10,000 km^2^. The reason behind this criteria is that due to the resolution of the satellite data and of the hydrological model, the performance of both is generally better for large and unregulated rivers. Although for there are some small rivers with good performance for broad and low gradient river systems. For this study, the remaining stations for validation are six for Africa and 95 for South America.

### 2.3 Satellite data

Remotely sensed surface water extent provided by the Global Flood Detection System (GFDS, http://www.gdacs.org/flooddetection/) was used for this study. This method uses a range of passive microwave sensors to calculate the difference in brightness temperature, at a frequency of 36.5 GHz, between water and land surface to detect the proportion of within-pixel water and land (Kugler and De Groeve, 2007). This dataset is available from 1998 until present, and during its life time, it has made use of different passive microwave sensors.

For our work and period of study, the merged Tropical Rainfall Measuring Mission (TRMM) and Advanced Microwave Scanning Radiometer for Earth Observation System (AMSR-E) product was used. However, GFDS is currently delivering a merged product of AMSR2 (http://suzaku.eorc.jaxa.jp/GCOM_W/w_amsr2/whats_amsr2.html) and the Global Precipitation Measurement (GPM, http://pmm.nasa.gov/GPM). The retrieved changes in brightness temperature are first gridded into a product with a pixel size of 0.09° × 0.09°, and then the system provides a daily output. In order to have a uniform grid size to match the hydrological model spatial resolution, we rescaled the GFDS data from 0.09° × 0.09° to 0.1° × 0.1°, around 10 km × 10 km (near the Equator) using a linear scaling method. We used the four day running mean provided by the GFDS operational product to avoid any missing days and occasional data errors which might provoke jumps in data typically lasting one to three days (Kugler and De Groeve, 2007).

### 2.4 Reference climatology forcing

The rainfall-runoff model was run using the WATCH Forcing Data methodology applied to the ERA-Interim data (WFDEI) meteorological dataset (Weedon et al., 2014), available from 1979 to 2013 at 0.5° by 0.5° spatial and daily temporal resolution. The WFDEI precipitation data were corrected using the gauge-based GPCPv2.2 dataset (Adler et al., 2003, Huffman et al., 2009, Huffman et al., 2012), provided by the eartH2Observe project (www.earth2observe.eu/). For surface albedo we used a monthly climatology based on the European Space Agency (ESA) GlobAlbedo product (Muller, 2013).

## 3 Methodology

This study integrates hydrological simulations of the LISFLOOD rainfall-runoff model (Section 3.1) for different scenarios (Section 3.2) with satellite-derived surface water extent through a data assimilation framework using the Ensemble Kalman Filter (Section 3.3) method. Validation of the simulated streamflow time series with, and without data assimilation is performed against ground streamflow measurements (Section 3.3.3).

### 3.1 Hydrological model: Global LISFLOOD

The LISFLOOD model is a distributed hydrological rainfall-runoff model that is capable of simulating the hydrological processes that occur in a catchment (Van Der Knijff et al., 2010). Originally developed for operational flood forecasting at European scale (Thielen et al., 2009), LISFLOOD has also been applied to assess the impact of climate change on floods (Alfieri et al., 2015, Rodrigo Rojas, 2013) and droughts (Forzieri et al., 2014) for Europe. For global ensemble streamflow forecasting and flood early warning (GloFAS) the model was set up on a global coverage with horizontal grid resolution of 0.1° (about 11 km in mid-latitude regions) and daily time step (Alfieri et al., 2013).

In this study, we used the version released in 2015 of the LISFLOOD Global model set up with some updates such as a fully modular, object-oriented python code, using the PCRaster (Karssenberg et al., 2010) python library, the possibility to use of netcdf files, and a data assimilation module. This new set up of the LISFLOOD model was used uncalibrated on this study, although it uses parameter values based on expert knowledge from previous runs of the LISFLOOD model. (Karssenberg et al., 2010) python library, the possibility to use of netcdf files, and a data assimilation module. This new set up of the LISFLOOD model was used uncalibrated on this study, although it uses parameter values based on expert knowledge from previous runs of the LISFLOOD model.

**Fig. 2:**
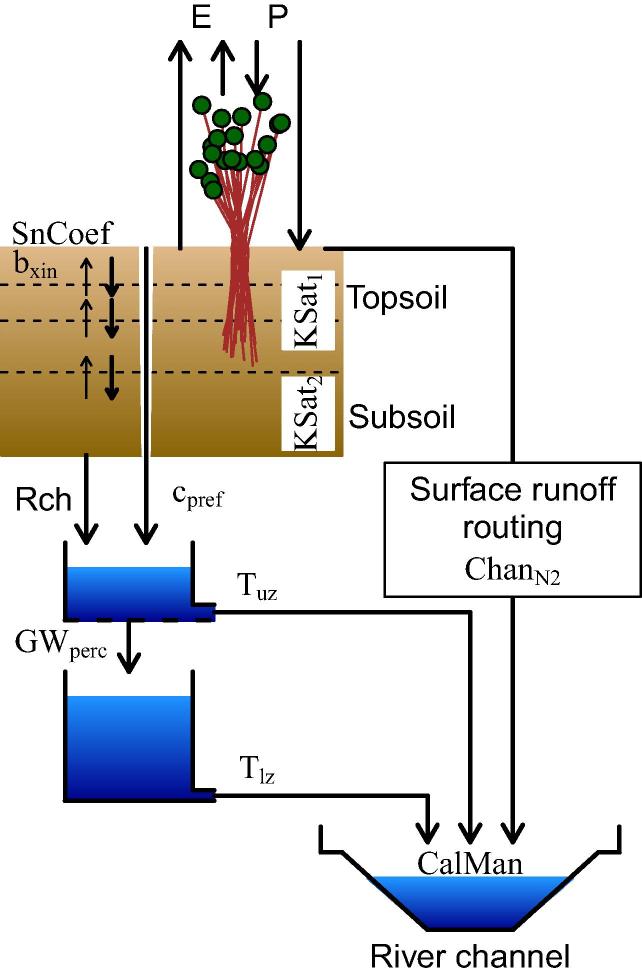
LISFLOOD Global model set up. Black arrows represent water fluxes; precipitation (P), evaporation (E), recharge from the unsaturated zone to the groundwater (Rch). The calibration parameters of the model are: snowmelt coefficient (SnCoef), Xinanjiang shape parameter (bxin), saturated conductivity of the topsoil (KSat2), empirical shape parameter preferential macro-pore flow (cpref), maximum percolation rate from upper to lower groundwater (Tlz), surface runoff roughness coefficient (ChanN2), and channel Mannings roughness coefficient (CalMan). The Xinanjiang parameter (bxin) is an empirical shape parameter in the Xinanjiang model (Zhao and Liu, 1995) that is used to simulate infiltration. It controls the fraction of saturated area within a grid cell that is contributing to runoff, hence it is inversely related to infiltration.

The model consists of a vegetation layer, two layers to simulate the unsaturated zone, two linear reservoirs to represent the fast and slow responding groundwater systems, and a channel network for streamflow routing. The processes simulated by the setup of the model (Fig. 2) include snowmelt, infiltration, interception of rainfall, leaf drainage, evaporation and water uptake by vegetation, surface runoff, preferential flow, and exchange of soil moisture between the soil layers (three topsoil and one subsoil layers). Wanders et al., 2014a, Wanders et al., 2014b replaced the original soil layer representation of the unsaturated zone by a new unsaturated zone model component of four layers, in order to use a first soil layer of 5 cm to compare with satellite derived soil moisture for data assimilation. Additional elements such as modelling of lakes and reservoirs behaviour, irrigation and water use were not included for this analysis, but they are currently available within LISFLOOD. Further information on the background of the LISFLOOD Global model and the description of the equations can be found in Burek et al. (2013).

### 3.2 Scenarios

To cope with the long memory of the groundwater storage component and to avoid unrealistic trends in the simulation it is necessary to calculate the average recharge rate into the lower groundwater zone based on a long term initialisation run (1979–2010). To initialise all the other storage components (e.g., snow cover, moisture content of the soil, upper groundwater zone storage, etc.) a relatively short warm up period (1 year) is sufficient in comparison to the long run needed due to the residence time in the system of the lower groundwater. This warm up run uses the average recharge rate into the lower groundwater zone. Result of this initial state run are store as state maps for every single day. These are the condition/state of all the internal 36 storage components and other conditions of LISFLOOD (e.g., soil moisture content of the first soil zone for forest land cover or frost index value).

Thereafter, three scenarios can be defined:

a. Deterministic simulation: using the daily state maps (e.g., snow cover, moisture content of the soil, upper groundwater zone storage, etc.), we run LISFLOOD in order to obtain the daily discharge maps and time series at predefined gauged sites (or gridded locations of interest).
b. Open-loop deterministic simulation: using the daily state maps, run LISFLOOD using the Monte Carlo approach by applying random sampling perturbations to the precipitation inputs to obtain probabilistic LISFLOOD simulations with 24 ensembles members in this case, but without data assimilation.
c. Data assimilation simulation: using the daily state maps, run LISFLOOD using the Monte-Carlo approach and the Ensemble Kalman filter (EnKF), to update state variable based on the assimilation of the satellite-derived surface water extent to obtain updated ensemble discharge maps and time series with the aim to improve the streamflow simulations with the information contained on the satellite-derived data. Further details on this process can be found on Section 3.3.

We applied random sampling perturbations the precipitation variable to account for uncertainty in the input data as precipitation is the main variable driven the simulation of the streamflow in tropical climate. All simulations used daily time steps for inputs and outputs time series.

### 3.3 Data assimilation

#### 3.3.1 Ensemble Kalman filter (EnKF) theory

The Kalman Filter (Kalman, 1960) is a sequential data assimilation method widely used in hydrological sciences. At each time step, new observations are combined with the model outputs derived from the simulated state (forecast) to compute an update state (analysis). This state is obtained by optimally taken into account observation and model errors. The original Kalman filter was extended for different schemes. Here, we used the Ensemble Kalman filter (EnKF) (Evensen, 1994, Evensen, 2003), a Monte-Carlo implementation of data assimilation using nonlinear models to propagate the ensemble states. The ensemble of model states is designed to represent model uncertainties, including those on the meteorological forcing, the model structure, and the parameters. To generate the ensembles, the precipitation forcing was scaled between 0 and 100 and perturbed with a white noise of mean of 1 and standard deviation of 0.15, to prevent ensemble deterioration. These values were chosen after testing as produced satisfactory perturbation results.

The general form of the EnKF (Evensen, 2003) is given as

(1) $$\Psi^{a}=\Psi^{f}+P^{f}H^{t}{\left({\mathrm{HP}}^{f}H^{t}+R\right)}^{-1}(d-H\Psi^{f})$$

where Ψ^a^ is the model analysis (updated Qmodel) and Ψ^f^ is the (deterministic) model simulation (Qmodel), P^f^ is the state error covariance matrix of the model, R is the error covariance measurements, and H is a measurement operator that relates the true model state Ψ^t^ to the (satellite) observations d, allowing for measurement error ε.

(2) $$d=H\Psi^{t}+\varepsilon$$

The error covariance matrix for the updated estimate P^f^ is define in the Kalman filter in terms of the true state as:

(3) $$P^{f}=\overset{‾}{(\Psi^{f}-\Psi^{t}){\left(\Psi^{f}-\Psi^{t}\right)}^{T}}$$

where the overline denotes an expectation value, Ψ is the model state vector at a particular time and the superscript ^f^ and ^t^ represent simulation and true state, respectively. However, the true stated is not known, and we therefore define the ensemble covariance matrix around the ensemble mean, $\overset{¯}{\Psi }$:

(4) $$P^{f}≃P_{e}^{f}\overset{‾}{=(\Psi^{f}-\overset{‾}{\Psi^{f}}){\left(\Psi^{f}-\overset{‾}{\Psi^{f}}\right)}^{T}}$$

#### 3.3.2 Ensemble Kalman filter (EnKF) within LISFLOOD

**Fig. 3:**
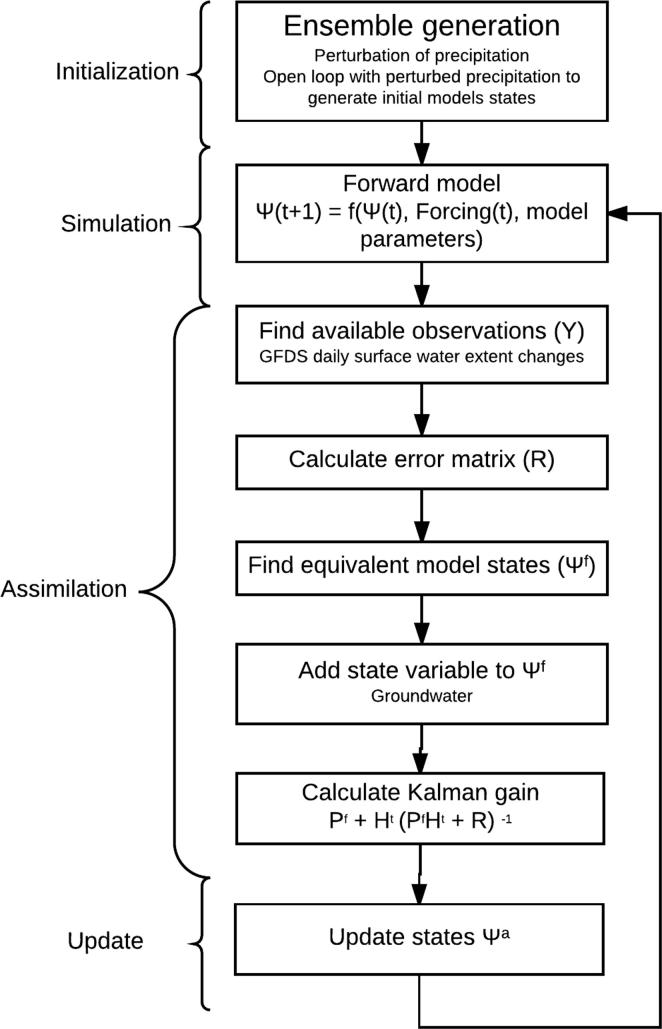
Flowchart of data assimilation scheme (based on Wanders et al. (2014a)).

In this study, the system state is simulated streamflow from LISFLOOD, while observations are given by satellite-derived surface water extent from GFDS. Data assimilation within the EnKF is performed at a daily time step which is identical to the temporal scale of the meteorological forcing, satellite observations, and the ground streamflow time series for a posterior validation. At each time step, the data assimilation scheme corrects the model according to the differences between simulated and observed streamflow volumes (Fig. 3). The model is corrected by using state augmentation to allow the simulated groundwater levels in the catchment to be updated instead of the simulated streamflow levels as the impact will last longer than for just assimilation of streamflow. The effect of updating the groundwater levels has a higher impact on the simulated flow than adjusting just streamflow levels, and it ensures a more stable streamflow signal. In order to reduce noise by potential erroneous measurements and computational demand, a threshold was applied to assimilate values: (a) between the 90th and 95th percentile of streamflow values for the entire continent and, (b) when the groundwater states in the upstream area were larger than zero (those avoiding assimilate if equal to zero).

The 90th to 95th percentile was based on values obtained by Revilla-Romero et al. (2014), to provide the most stable and realistic observations for large-scale rivers and is therefore applied in this study. In order to limit computation cost, we use 24 ensemble members and obtain stable results. Afterwards, the simulated streamflow with EnKF was compared to a simulated run without data assimilation (baseline), the deterministic, and open-loop run. We acknowledge that daily simulations will not be able to capture sudden changes in water levels driven by heavy rain events. Subdaily simulations will be more appropriate given precipitation inputs, remote sensing and validation datasets are available.

The error covariance between the streamflow observations is set to zero while the standard error for the streamflow observation is assumed to be 30% of the actual discharge (Di Baldassarre and Montanari, 2009). Further, the covariance between the satellite surface water extent and discharge observations was set up to zero. As the measurement error variance (R) is needed within the EnKF assimilation method, we assumed that the spatial standard error of the satellite observations or average standard error is 1030.40 m^3^ s^−1^. The error was calculated using all stations from Revilla-Romero et al. (2014). This is the best estimate of the GFDS signal currently available and has therefore been used in this study. Tests made during this study suggested that a variable error depending on the river volume at each location (percentile error) might be more appropriate and needs further research.

In addition, the GFDS observations are bias-corrected, using a linear bias correction method that is constrained between the minimum and maximum values of the historically simulated discharge values. A linear bias correction was implemented to ensure that the satellite derived data keep their original distribution and the distribution is not biased by erroneous discharge simulations. Moreover, the linear interpolation, ensure that the distribution remains identical to raw observations and does not require observations to estimate the empirical distribution. Finally this bias correction method also ensures that the implemented EnKF can be used in other locations without the need for ground observations.

Mathematical framework:

Linear rescaling of the dimensionless satellite surface water changes observations:

(5) $$\text{Qobs}=\left(\frac{\text{Obs}-\text{MinObs}}{\text{MaxObs}-\text{MinObs}}\right)\times (\text{MaxMod}-\text{MinMod})+\text{MinMod}$$

where Obs are the satellite observation, and Mod are the modelled streamflow values.

(6) $${\text{Threshold}}_{\text{min}}=[90\%{\text{Q}}_{\text{obs}}]\text{;}\;{\text{Threshold}}_{\text{max}}=[95\%{\text{Q}}_{\text{obs}}]$$

If Threshold_min_ < Q_obs_ & Q_model_ < Threshold_max_ then the groundwater levels from upper (UZ) and lower (LZ) are updated.

(7) $$\text{Covariance matrix}={\text{QMod}}^{\text{satError}}$$

where satError is the best available estimate of the GFDS spatial standard error or average standard error signal currently available.

#### 3.3.3 Assessing the value of GFDS though data assimilation

We carried out a comparison of the streamflow time series with and without assimilation of satellite-derived surface water extent observations. In addition, we also evaluated the model performance against the ground streamflow observations. For this, we used some of the more commonly used metrics in hydrology: the coefficient of determination (R^2^), Kling-Gupta Efficiency (KGE’), root-mean-square error (RMSE), volumetric efficiency (VE), the Nash-Sutcliffe Efficiency (NSE) and percentage of bias (PBIAS%).

First, the coefficient (R^2^) was calculated, which values ranges from −1 to 1, with 1 being the optimum value. Second, we used the modified Kling-Gupta Efficiency (KGE’; Kling et al., 2012) as a performance indicator based on the equal weighting of linear correlation (r), bias ratio (β) and variability (γ), between simulated (s) and observed (o) streamflow values:

(8.a) $$\mathrm{KGE}’=1-\sqrt{{\left(r-1\right)}^{2}+{\left(\beta -1\right)}^{2}+{\left(\gamma -1\right)}^{2}}$$

(8.b) $$\beta =\frac{\mu_{s}}{\mu_{o}}$$

(8.c) $$\gamma =\frac{{\mathrm{CV}}_{s}}{{\mathrm{CV}}_{o}}=\frac{\sigma_{s}/\mu_{s}}{\sigma_{o}/\mu_{o}}$$

where CV is the coefficient of variation, μ is the mean streamflow [m^3^ s^−1^] and σ is the standard deviation of the streamflow [m^3^ s^−1^]. KGE’, r, β and γ have their optimum at unity. The KGE’ measures the Euclidean distance from the ideal point (unity) of the Pareto front and is therefore able to provide an optimal solution which is simultaneously good for bias, flow variability, and correlation.

The root-mean-square error (RMSE) also measures the differences between values predicted by a model and the values actually observed:

(9) $$\mathrm{RMSE}=\sqrt{\frac{\sum_{t=1}^{T}{\left(Z_{\mathrm{mod}}(t)-Z_{\mathrm{obs}}(t)\right)}^{2}}{n}}$$

where Z_mod_ is the modelled ensemble mean streamflow, Z_obs_ is the observed streamflow, T is the total number of time steps, and n is the total number of observation. In order to compare streamflow time series of different gauge stations, the RMSE was standardised on the average streamflow of each station, Q_obs_:

(10) $$\mathrm{CV}(\mathrm{RMSE})=\frac{\mathrm{RMSE}}{\overset{‾}{Q_{\mathrm{obs}}}}$$

The volumetric efficiency was proposed in order to circumvent some problems associated to the Nash-Sutcliffe Efficiency. It ranges from 0 to 1, and represents the fraction of water delivered at the proper time; its compliment represents the fractional volumetric mismatch (Criss and Winston, 2008).

(11) $$\mathrm{VE}=1-\frac{\Sigma |{\text{Q}}_{\text{sim}}-{\text{Q}}_{\text{obs}}|}{\Sigma ({\text{Q}}_{\text{obs}})}$$

For the Nash-Sutcliffe and percentage of bias metrics, please refer to the Appendix.

## 4 Results and discussion

**Fig. 4:**
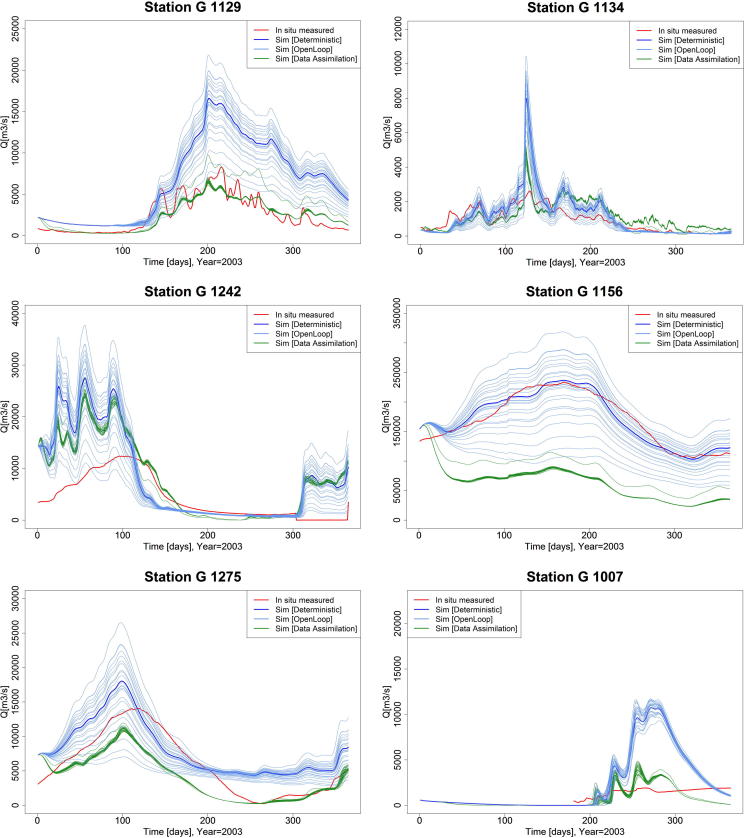
Comparison of daily LISFLOOD simulated [Deterministic, Open Loop and Data Assimilation] runs and *in situ* observed hydrograph during 2003, for (a) Branco River at Caracarai [G1129], (b) Araguari River at Porto Platon [G1134], (c) Araguaia River at Conceicao do Araguaia [G1242], (d) Amazon River at Obidos-Linigrafo [G1156, (e) Rio Mamore at Guajara-Mirim [G1275], and (f) Niger River at Niamey [G1007].

Fig. 4 shows the comparison between simulated (Deterministic, Open Loop, and Data Assimilation) and *in situ* observed hydrographs for six selected locations for the year 2003. A distinct spatial pattern was found for the improvements in the LISFLOOD simulations after data assimilation (DA). This pattern is most likely caused by the differences in the signal-to-noise ratio of the GFDS signal, leading to a lower quality. At some locations, the improvement in streamflow simulations after DA is significant in both the timing and volume of the flow peaks (e.g., station G1129, G1134, G1242), at other locations, such as on the Amazon River, the DA resulted in a major underestimation of the simulated streamflow time series. An additional factor affecting the potential for improvement after DA with GFDS observations, is the AMSR-E signal which is hampered over densely vegetated areas (de Jeu et al., 2008, Njoku et al., 2003, Parinussa et al., 2011, Wanders et al., 2012). This could have a significant impact on the GFDS signal, leading to lower signal-to-noise ratios. For example, for the station Obidos Linigrafo (G1156, see Fig. 4) on the Amazon River, we argue that the deterioration of the simulated streamflow after Data Assimilation is potentially driven by these two problems. On one side, even though the GFDS is able to capture well the timing of the highs and lows on the Amazon River, the day to day raw signal-to-noise ratio is rather low, resulting in a “noisy” signal and a low variability of the signal. This is corroborated by a previous calibration study (see Revilla-Romero et al., 2015, Fig. 3) where the raw GFDS signal was used to enhance the timing of the simulated streamflow. Note that a different meteorological forcing and set up of the LISFLOOD model was used in that study, and it was only calibrated using the correlation and not explicitly in terms of volume.

On the other side, due to the large volume of rivers such as the Amazon, we found out that the error covariance measurement approach used might not have been appropriate at these locations deriving on a high streamflow underestimation. The best available measurements to calculate the error covariance was based on the mean RMSE error calculated from all the studied stations (Revilla-Romero et al., 2014), which as seen here is not representative for all locations. In the current approach, the cross covariance between GFDS observations is not accounted for due to absence of reliable data hampering the estimation of the spatial error structure.

**Fig. 5:**
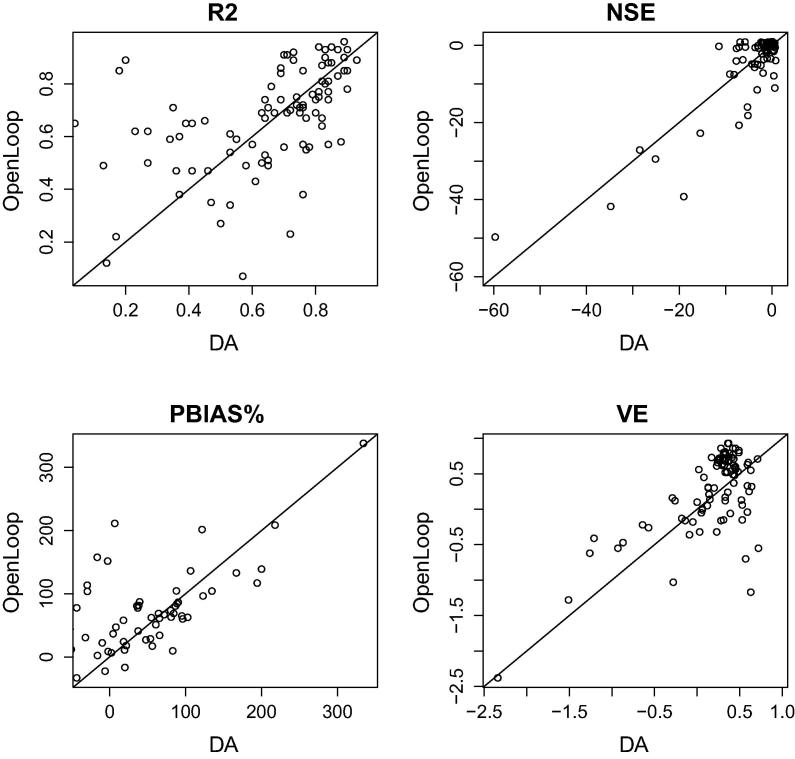
Differences obtained by the mean ensemble of the Open Loop and of the Data Assimilation (DA) run on six scores for all South American and African river gauge stations.

We carried out a comparison of the mean of the 24 ensemble members of simulated streamflow with Data Assimilation of daily satellite-derived surface water extent observations and with perturbations of the deterministic run (Open Loop). The scores are based on the skill of each simulated run to represent the characteristics of the *in situ* observation time series at each location. The hydrological performance on the studied period is illustrated in Fig. 5. Results are evaluated based on six metrics: R^2^, NSE, PBIAS% and VE. In terms of the R^2^ there are a mix of results, therefore the data assimilation framework employed does not show a big potential in this case. We found that when measuring the NSE, PBIAS%, and VE, the largest improvement is achieved at those locations where the Open Loop simulation is poorer. For example, looking at the NSE skill scores, an improvement was obtained after DA for 61 out of 101 stations.

**Fig. 6:**
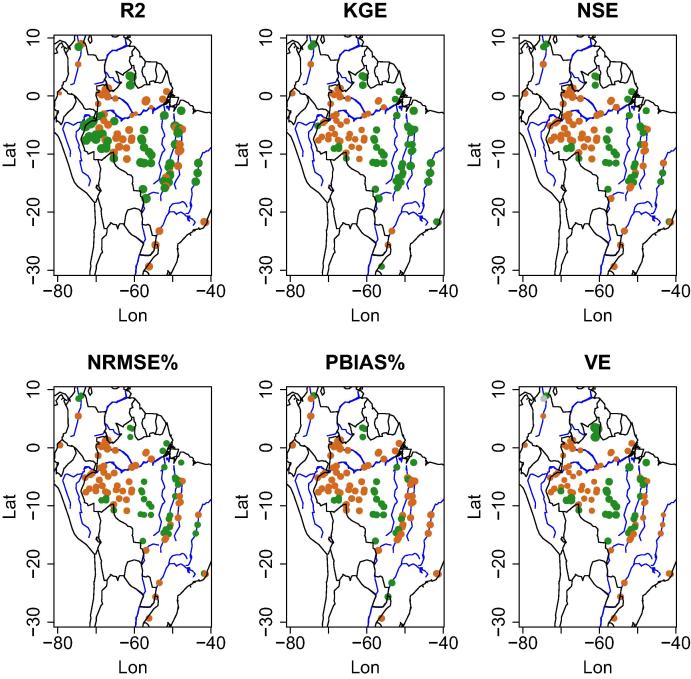
Differences obtained by the mean ensemble of the Open Loop and of the Data Assimilation (DA) run on six scores for South American river gauge stations. Green dots represent the stations where the DA run enhanced the simulation of the streamflow, whereas the brown dots show where the skill decreased. Dot size represents the percent difference after DA. (For interpretation of the references to colour in this figure legend, the reader is referred to the web version of this article.)

**Fig. 7:**
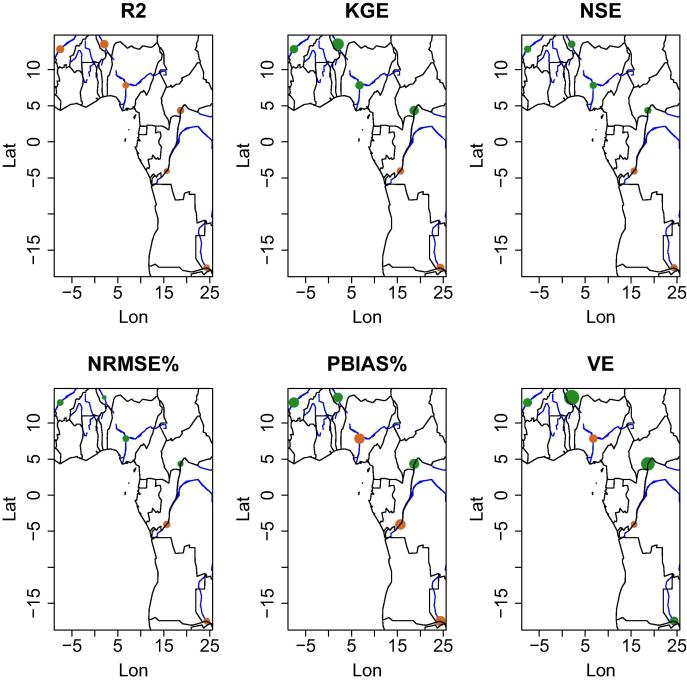
Differences obtained by the mean ensemble of the Open Loop and of the Data Assimilation (DA) run on six scores for African river gauge stations. Green dots represent the stations where the DA run enhanced the simulation of the streamflow, whereas the brown dots show where the skill decreased. Dot size represents the percent difference after DA. (For interpretation of the references to colour in this figure legend, the reader is referred to the web version of this article.)

Figs. 6 and 7 show the differences obtained by the mean ensemble of the Open Loop and the Data Assimilation runs for the South American and African stations. We found out that there is a clear spatial pattern on the performance using the satellite signal for data assimilation. Overall, for all metrics, data assimilation leads to a decline in performance for most of the stations in the lowland jungle of the Amazon basin; whereas, at stations in other (sub)basins DA leads to an improvement. There are a number of factors which affect the skill of the satellite signal in retrieving surface water changes. For example, closed forest, such as the tropical rainforest, has an effect on the quality of the data retrieved for the satellite (as discussed before) and this tends to influence the signal-to-noise retrieved from the stations located on the main Amazon river.

Ultimately, this framework could be applied and tested within an ensemble hindcasting procedure to evaluate the potential of the satellite-derived surface water changes within a forecasting system. However, before that a number of steps need to be implemented. The covariance error should be further investigate to reach an optimum value for each location. This could be done by calculating a relative error depending on the simulated streamflow volume at each location instead of applying a single value for all. By doing so, we will expect a better prediction skill of the DA scenario. Recently, Van Dijk et al. (2016), studied the feasibility of using satellite water extent, from GFDS and MODIS, to derive satellite water discharge on over 8000 gauging stations. Conclusions on the different performance across locations and driven by the local conditions agrees with the findings in Revilla-Romero et al. (2014). Further research should be done to understand the observation error at each (ideally ungauged) location on river streams around the globe.

Furthermore, it could be investigated an alternative method to avoid the worsening of the simulated discharge by, for example applying a more stricter quality criteria for assimilating GFDS signal. Next, it is important to remark that the validation was carried out at gauge points of the catchments whereas the assimilation was done using the data available for the full continent, as the GFDS product is available at the (near-) global scale on a daily basis. This could be another reason of the poor results at some locations. It could be tested to assimilate the GFDS on the pixels along river reaches, excluding those that purely contain land features. Another approach that we could have taken is to assimilate either the raw GFDS signal only at the locations where we have gauges for validation, or to assimilate GFDS estimated streamflow as done by Zhang et al. (2013) at one location. However, previous historical streamflow measurements are needed to derived these streamflow estimations. All these tests could be done independently of the rainfall-runoff model used for data assimilation of the GFDS signal, as long as using the EnKF methodology. In fact, it will be recommendable to test this data assimilation framework for other models different than LISFLOOD to better understand the potential with different base or deterministic prediction skills.

In addition, as both the satellite and the meteorological forcing time series are available for a longer period (e.g., the GFDS data from 1998), it would be beneficial to apply the data assimilation framework over an extended period of time. Even though the assimilation of ground streamflow data may improve the simulation outputs (e.g., Clark et al., 2008, Lee et al., 2011, Moradkhani et al., 2005, Wanders et al., 2014a), in this study we did not incorporate these observations as the aim was to solely assess the impact of assimilating surface water extent from the GFDS system.

## 5 Conclusion

The limited availability of continuous and up-to-date ground observational data is one of the main constraints for real-time applications such as global flood forecasting models. This work was designed to assess the value of using satellite retrieved surface water extent changes from the Global Flood Detection System (GFDS) to improve hydrological modelling simulations. The gain from assimilation of GFDS signal is compared to the current simulation of the LISFLOOD model with a Monte Carlo (Open Loop) approach to derived ensemble members, for a one-year period, in station in South America and Africa. The main conclusions of the study are summarized as follows:

(1) As a proof-of-concept study, the results presented here show the possibility of using surface water extent changes from GFDS within an EnKF data assimilation framework for the rainfall-runoff LISFLOOD model. This framework was validated in over 100 *in situ* gauges. Largest gain on reproducing *in situ* streamflow was obtained by the locations with poorest skill scores on the deterministic runs, as in Branco River at Caracarai [G1129]. For example, NSE score improved on 61 out of 101 stations. In order to improve the performance of the data assimilation framework, it should also be tested to include the GFDS data at places with high variability and signal to noise ratio, and to calculate a relative error depending on the simulated streamflow volume at each location instead of applying a single value for all studied locations.
(2) Due to the low signal-to-noise ratio present at some locations (Revilla-Romero et al., 2014), for example, located in an area where the predominant land cover is closed forest (lowland jungle), the application of these data within a data assimilation technique might not be beneficial for all locations, such as at stations located on the main Amazon river. However, benefits were found on other locations situated in non-closed forest.
(3) In order to make the most out of the information provided on a daily basis by the GFDS satellite signal, within a data assimilation framework, the calculation of the covariance error should be further investigate to reach the optimum at the largest amount of studied location. We confirm that the covariance error has an important role on the outputs from the assimilation framework.

## Figures and Tables

**Table 1:** Summary of relevant studies where satellite-derived data was assimilated within a hydraulic and/or hydrological model. Studies are listed in alphabetical order author.

	Study	Satellite and sensor/Acquisition frequency	Model	DA method	Study area and no. of *in situ* river gauges used	Study period	Objective/Approach	Key findings
1	Andreadis et al. (2007)	Synthetic Water Surface Level/8 days	LISFLOOD-FP	Ensemble Kalman Filter (EnKF)	50-km reach, Ohio River (USA)	01 April to 23 June 1995	Synthetic surface water elevation profiles produced to assimilate within a hydraulic model	The filter successfully recover water depth and discharge from a corrupted LISFLOOD-FP simulation

2	García-Pintado et al. (2013)	Synthetic Aperture Radar (SAR, COSMO-Skymed Water Levels)	LISFLOOD-FP	ETKF	Lower Severn and Avon rivers (UK)	19 July to 01 August 2007	Evaluate the forecast sensitivity to satellite first visit and revisit time	Online correction of imposed bias clearly improves the 2D flood model/DA forecast. Revisit interval is most influential for early observations
3	Giustarini et al. (2011)	ERS-2 SAR and ENVISAT ASAR (WL)	HEC-RAS	Particle Filter	19 km reach of the Alzette River (Luxembourg)	January 2003	Integration of water level data into an one-dimensional (1-D) hydraulic model	The updating of hydraulic models through the proposed scheme improves model predictions over several time steps

4	Hostache et al. (2010)	SAR (RADARSAT-1) (WL)	Shallow water equations (2D-SWEs)	Variational data assimilation (4D-var)	28 km reach of the Mosel River (France/Germany) (3 gauges)	28 February 1997	Assimilation of satellite-derived water levels in a 2D shallow water model	DA enhances model calibration, optimal to identify Manning friction coefficients in the river channel

5	Mason et al. (2012)	SAR (TerraSAR-X)	Hydraulic model (not specified)	Not specified	Lower Severn and Avon rivers (UK) (2 gauges)	19 July to 01 August 2007	Development of a methodology to employ waterline assimilation to correct the model state	Waterline levels from SAR images may be assimilated. The levels extracted from a SAR image of flooding agreed with nearby gauge readings

6	Matgen et al. (2010)	SAR (WL)	Coupled Hydrologic-Hydraulic (H-H)	Particle Filter	19 km reach of the Alzette River (Luxembourg)	01 to 07 January 2003	Development of a new concept for sequential assimilation of SAR-derived water stages into coupled H-H models	Significant uncertainty reduction of water level and discharge at the time step of assimilation

7	Munier et al. (2015)	SWOT/21 days (Virtual data)	VIC + LISFLOOD-FP	EnKF	Upper Niger River Basin (Africa) (4 gauges)	July 1989 to June 1990	EnKF is used to assimilate SWOT data into a coupled hydraulic reservoir model	The persistence of the assimilation greatly increases with the use of a smoother

8	Pedinotti et al. (2014)	SWOT/1 or 3-day subcycle (Virtual data)	ISBA-TRIP	Extended Kalman Filter (EKF)	Niger River Basin (8 gauges)	June 2002 to 2003	Study the impact of assimilating SWOT observations to optimise Manning’s roughness coefficient	Demonstration SWOT’s promising potential for global hydrology issues

9	Lai et al. (2014)	MODIS flood extent (250 m)/Daily	2-D flood model (not specified)	4D-Var	Huaihe River, flood detention area (180 m^2^) (1 gauge)	29 June to 15 July 2007	Direct assimilation of the flood-extent data into a 2-D flood model. A 4D-Var method incorporated with a new cost function is introduced	Promising way of data assimilation for flood inundation modelling by using directly flood extent suitable for improving flood modelling in the floodplains or similar areas

10	Zhang et al. (2013)	GFDS (AMSR-E) + TRMM rainfall + WL/Daily	HyMOD	Ensemble Square Root Filter (EnSRF)	Cubango River Basin (1 gauge)	2003–2005	Investigate the utility of satellite data estimates in improving flood prediction	Shows opportunities in integrating satellite data in improving flood forecasting by careful fusion of remote sensing and in-situ observations

11	This study	GFDS (AMSR-E + TRMM) flood extent/Daily	LISFLOOD	EnKF	African (6 gauges) and South America Continent (95 gauges)	2003	Test the impact of satellite-derived daily surface water extent in continental hydrological modelling	Assess the potential of assimilation of GFDS data into large scale hydrological model. Largest were obtained by the locations with poorest skill scores on the deterministic runs. However, it might not be beneficial for all locations
